# Parental obesity predisposes male and female offspring to exacerbated cardiac dysfunction and increased mortality after myocardial infarction

**DOI:** 10.1152/ajpheart.00827.2024

**Published:** 2025-03-20

**Authors:** Jussara M. do Carmo, Ana C. M. Omoto, John E. Hall, Xuemei Dai, Emily C. Ladnier, Marilia C. Mouro, Odecio E. S. Tosta, Zhen Wang, Xuan Li, Alexandre A. da Silva

**Affiliations:** 1 Department of Physiology and Biophysics, Mississippi Center for Obesity Research, Cardiorenal and Metabolic Diseases Research Center, University of Mississippi Medical Center, Jackson, Mississippi, United States; 2 Faculdade de Medicina, Centro Universitário Barão de Mauá, Ribeirão Preto, Brazil

**Keywords:** arrhythmia, connexin 43, heart failure, mitochondrial function, mortality rate

## Abstract

Acute myocardial infarction (MI) is a leading cause of death worldwide, accounting for >1 million deaths/year in the United States alone. Although parental obesity is a risk factor for offspring cardiovascular diseases, the impact of parental obesity on offspring outcomes after MI is unknown. This study examined if non-obese male and female offspring from obese Sprague-Dawley rat parents fed a high-fat diet (HFD-Offs, *n* = 11–19/sex) are at greater risk of death and worse cardiac dysfunction after MI, compared with offspring from lean parents fed a normal diet (ND-Offs, *n* = 12–15/sex). All offspring were fed ND from weaning and subjected to left descending coronary artery ligation at 12 wk of age to induce MI. Survival rate 24 h post-MI was examined, and cardiac function was measured by echocardiography and intraventricular catheterization with a Millar catheter on *day 7* post-MI. Compared with ND-Offs, male and female HFD-Offs exhibited increased ventricular fibrillation and reduced survival post-MI (male: 37% vs. 80% and female: 55% vs. 83% for HFD-Offs and ND-Offs, respectively). In surviving rats, systolic dysfunction was more pronounced in male and female HFD-Offs compared with ND-Offs at *day 7* post-MI, despite similar infarct size in all groups. We also found reductions in baseline O_2_ consumption rate and pyruvate-supported mitochondrial respiration, as well as increased mitochondria-derived superoxide production in cardiac fibers from HFD-Offs. Thus, parental obesity is associated with an increased 24-h mortality rate in their offspring after induction of MI and worse systolic function even when the offspring are fed a healthy diet after weaning and remain lean.

**NEW & NOTEWORTHY:**

A major new finding of this study is that parental obesity markedly reduces survival rate and exacerbates cardiac dysfunction after myocardial infarction in their offspring, and this effect is independent of offspring sex.

## INTRODUCTION

Obesity is linked with numerous cardiovascular diseases, including atherosclerosis, heart failure, and arrhythmias, particularly sudden cardiac death and atrial fibrillation (1, 2). Although obesity itself heightens the risk of metabolic derangements and cardiovascular diseases, the rising proportion of men and women at reproductive ages who are overweight/obese may have profound consequences for future generations since parental obesity appears to program their offspring for obesity and associated cardiometabolic diseases.

Previous studies in humans and rodents show that maternal obesity is associated with a greater risk of metabolic disorders in the offspring (3, 4). For instance, the HUNT study showed that maternal overweight in humans is strongly associated with offspring overweight (5), and data from the Framingham Heart Study suggest that offspring from obese parents have a higher risk of developing obesity, systemic inflammation, and altered neurohormonal activity (6). Metabolic abnormalities have also been reported in offspring from obese mice even when the offspring are fed normal diets and do not develop obesity (7–9). In addition to metabolic abnormalities, clinical and experimental studies also suggest that maternal obesity may adversely affect fetal myocardial function. For example, Ingul et al. (10) observed altered cardiac function in fetuses of pregnant women with obesity, including a ~33% reduction in the left (LV) and right ventricle’s global strain rate and strain, as early as the first trimester of gestation, and a ~27% thicker interventricular septum in late gestation when compared with fetal hearts from lean control pregnancies. Bayoumy et al. (11) also observed impaired LV fetal strain and global strain rate during the third trimester of gestation in obese and diabetic mothers compared with lean controls.

Using a model of parental obesity with male and female Sprague-Dawley rats fed a high-fat diet (HFD) before mating and dams maintained on the HFD during gestation and lactation, we found that male and female offspring from these obese parents have multiple metabolic abnormalities early in life (3 wk of age) (12). We also found that 3-wk-old male, but not female, offspring from obese parents show impaired diastolic function associated with reduced cardiac sirtuin 3 (SIRT3) levels and mitochondrial biogenesis (12). In addition, adult male, but not female, offspring from obese parents exhibited increased blood pressure compared with lean controls (13). These observations, combined with the fact that parental obesity programs metabolic disorders including insulin resistance in their offspring, raise the possibility that parental obesity may increase offspring susceptibility to worse cardiovascular outcomes later in life, especially when the offspring are subjected to additional stresses.

To test this hypothesis, we examined whether parental obesity programs cardiac dysfunction in their offspring and increases offspring susceptibility to exaggerated adverse cardiac remodeling and dysfunction, including higher mortality, in response to acute myocardial infarction (MI), even when the offspring consume a healthy diet and remain lean. We also investigated potential sex differences in cardiac function post-MI in offspring from obese and lean parents. Our results indicate that parental obesity is associated with more profound systolic dysfunction and increased mortality when their nonobese offspring are submitted to acute MI and that this worse outcome is independent of the sex of the offspring.

## METHODS

All experimental protocols and procedures conformed to the National Institutes of Health Guide for the Care and Use of Laboratory Animals and were approved by the Institutional Animal Care and Use Committee of the University of Mississippi Medical Center, Jackson, Mississippi. Rats were placed in a 12-h dark (6:00 PM to 6:00 AM) and light (6:00 AM to 6:00 PM) cycle and given free access to food and water throughout the study.

### Animals

Experiments were performed on 12-wk-old male and female offspring of Sprague-Dawley rats maintained at the animal facilities at the University of Mississippi Medical Center.

Parental obesity was induced by feeding sires and dams a high-fat diet (HFD, Western diet, TD.08811—45% fat, 15% protein, and 40% carbohydrate: 4.7 kcal/g) from 4 wk of age until the end of lactation (with mating starting at 9–12 wk of age). Lean control parents were fed a normal diet (ND—17% fat, 29% protein, and 54% carbohydrate: 3.0 kcal/g) and mated at the same age as the obese breeding pairs. We used 7–10 breeder pairs with litter sizes of 8–10 pups/mother, with only 1–2 pups/mother used for each experiment. Male and female offspring from obese and lean parents from our colony were weaned at 21 days of age and fed ND for the entire study period. The offspring were maintained on an ND to separate any potential effects of parental programming from the adverse effects of an obesogenic diet in the offspring.

### Surgical Procedures

**Figure 1.**
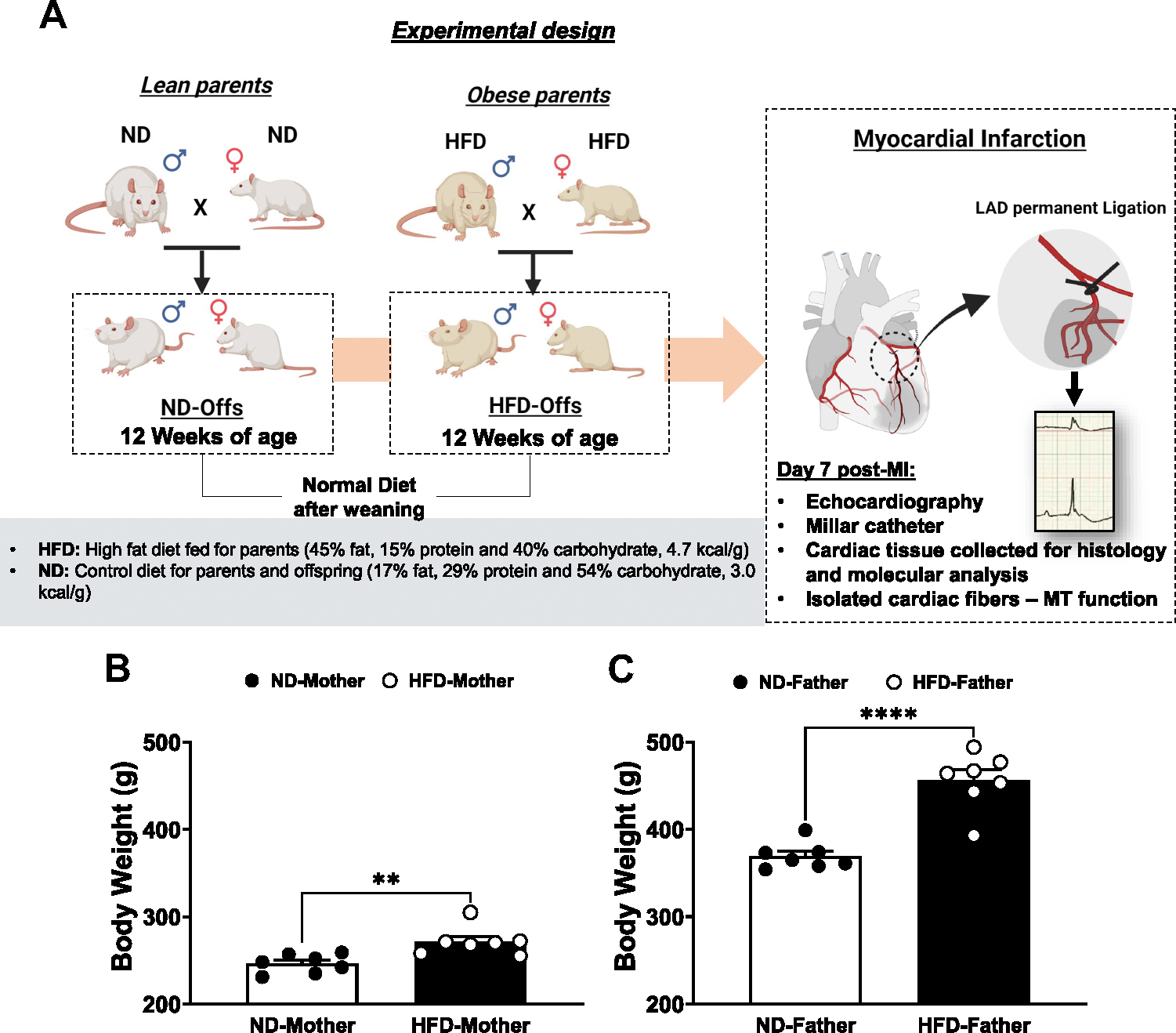
Experimental design and male and female parental body weight. *A*: off-spring from lean (fed a normal diet, ND) or obese parents (fed a high-fat diet, HFD). All offspring were fed an ND after weaning and were submitted to myocardial infarction (MI) at 12 wk of age. *B* and *C*: body weights of mothers and fathers fed an ND or HFD at 15–16 wk of age (n = 7 male and female breeders/group, including breeders whose offspring died after MI surgery). **P < 0.01 vs. ND-mother, ****P < 0.0001 vs. ND-father, unpaired two-tailed *t* test. MT, mitochondria; LAD, left anterior descending coronary artery.

Rats were anesthetized with isoflurane (2%), and endotracheal intubation was conducted using size 90 polyethylene tubing that was connected to a mechanical ventilator (Harvard Apparatus), as previously described (14). The thoracic area was shaved and disinfected with iodine and 70% ethanol, an incision was made at the fourth left intercostal space, and the chest was opened using a chest retractor between the ribs followed by pericardium removal; then, the left anterior descending coronary artery (LAD) was permanently ligated using 4–0 prolene suture (Ethicon). The chest retractor was removed, the ribs were drawn together, the muscle layers were carefully sutured, and negative thoracic pressure was restored by inserting a syringe needle into the chest cavity and aspirating air until significant resistance to air aspiration was observed, suggesting that negative pressure has been restored. This procedure was performed although the animal was under ventilatory support, immediately after suturing the muscles and closing the thoracic cavity, and then the skin was closed. Antibiotic (penicillin G benzathine) was applied to the wound just before closure of the skin, and buprenorphine (0.1 mg/kg sc) was administered immediately after surgery and again 24 h later. The effectiveness of the surgical procedure to induce MI was verified by ST segment elevation on electrocardiogram (Fig. 1).

We used ECG monitoring to assess potential cardiac arrhythmias, starting a few minutes before LAD ligation and continuing until the animal recovered from anesthesia. We visualized whether ventricular arrhythmias, including ventricular fibrillation (VF), occurred during recovery from anesthesia. If VF was observed, the investigator massaged the heart in an attempt to rescue the heart from VF and to avoid death.

### Echocardiography

Male and female offspring were anesthetized with isoflurane (1%–2%) and placed on a heating table, and their extremities were fixed to four electrocardiography leads on *day 7* after LAD ligation. Systolic function was determined by acquiring parasternal long-axis B-mode images at maximum left ventricular length (including visible apex, aortic valve, and the aorta) using the VisualSonics—Vevo 3100 system preclinical ultrasound system, 21-MHz transducer MX250 at 100 frames/s. Averaged (3–4 cycles) left ventricle (LV) tracings at systole and diastole of the same cardiac cycle were used to calculate ejection fraction (EF), fractional shortening (FS), and stroke volume (SV) parameters using VevoLab v. 3.2.6 software. EF was calculated using LV tracings in the parasternal long-axis B-mode according to the formula LVEF = [(LVEDV — LVESV)/LVEDV] × 100, automatically calculated using the VevoLab v. 3.2.6 software, and SV was calculated based on LV volume changes. The same parasternal long-axis B-mode images were used to calculate LV global longitudinal strain using the VevoStrain modality of the VevoLab software by manually tracing the endocardial and epicardial layers during the end-systolic or end-diastolic periods; after which the software identifies tissue speckles by tracking the movement of the ventricular wall in a frame-by-frame mode through the cardiac cycle. Diastolic function was also assessed at baseline, before MI surgery was performed, using the apical four-chamber view in pulsed-wave (PW) and tissue Doppler modes at the mitral valve level. The diastolic parameters assessed were mitral valve early peak velocity (MV E) divided by mitral valve tissue annular displacement e′ (E/e′) and isovolumetric relaxation time (IVRT) from 5 to 7 cardiac cycles. Animals that did not develop LV anterior wall akinesia on *day 7* post-MI surgery were excluded from the study.

### Experimental Protocol

Male and female offspring from obese and lean parents were weaned at 21 days of age and fed ND for the entire protocol (Fig. 1A). The offspring were derived from female and male breeders (~14 wk of age at the time of pregnancy) that were fed a normal diet (ND) or a high-fat diet (HFD) (Fig. 1, B and C). At 12 wk of age, the offspring’s baseline cardiac function was assessed by echocardiography, and permanent ligation of the left anterior descending (LAD) coronary artery was performed to induce MI. The surgical table contained ECG leads and a monitor with a homeothermic heating element to examine ventricular arrhythmias including VF after LAD ligation. Twenty-four-hour survival rate post-MI surgery was examined, and cardiac function was assessed on *day 7* post-MI when heart tissue was collected for molecular and histological analysis (Fig. 1A). All data analyses were performed by a blinded investigator.

### LV Pressure Measurements

At the end of the protocol, the rats were anesthetized with urethane (1 g/kg) and placed on a temperature-controlled heating pad to maintain body temperature. A pressure catheter (Millar 1.4 F, SPR 838, ADInstruments, New Zealand) was connected to a Mikro-Tip Pressure Volume System Ultra unit and a Power Lab digital data acquisition system (ADInstruments, New Zealand) and inserted into the LV through the right carotid artery, and ventricular pressure was recorded for at least 10 min. The following parameters were calculated: maximal rate of LV pressure increase (d*P*/d*t*_max_) and decrease (d*P*/d*t*_min_), and isovolumetric relaxation time constant (τ).

Euthanasia was performed using isoflurane overdose followed by pneumothorax in compliance with our IACUC-approved guidelines.

### High-Resolution Respirometry in Isolated Cardiac Fibers

Cardiac fibers from the LV septal area were isolated, weighed, and permeabilized with saponin and used to measure carbohydrate (pyruvate) and lipid (palmitoyl)-supported mitochondrial respiration rates. The respiration medium (MIR05) was equilibrated with oxygen (O_2_) gas to an initial dissolved concentration of 600 μM in the two-chamber titration-injection respirometer (Oxygraph2-k, Oroboros Instruments, Austria) and sealed. High-resolution respirometry was combined with the fluorescent sensor of the O2K-Fluo LED2 module for hydrogen peroxide (H_2_O_2_) production (Amplex Ultrared 10 μM, Invitrogen, 1 U/mL horseradish peroxidase, and 5 μ/mL superoxide dismutase). The respiratory control ratio was used as a quality assurance marker and cytochrome C (10 μmol/L) as a validation of proper tissue preparation.

Pyruvate (5 mM) and malate (2 mM) were added to one chamber to supply the electron transport chain via reduced intermediates generated in the citric acid cycle, and palmitoyl carnitine (5 μM) and malate (2 mM) were added to the other chamber to supply substrate for mitochondrial β-oxidation. Then ADP was added, followed by glutamate (3 mM) and succinate (6 mM) to support electron flux to complexes I and II to measure maximal oxidative phosphorylation capacity in both chambers. ATP synthase activity was blocked by oligomycin (4 μg/mL) to quantify leak-state oxygen consumption. Carbonyl cyanide 4-trifluoromethoxy phenylhydrazone (FCCP) was added in the chambers to measure uncoupled oxygen consumption. Respiration rates were corrected to fresh tissue mass.

### Western Blot Analysis

Heart samples from additional groups of male and female offspring from lean and obese parents with no MI surgery were homogenized in lysis buffer (KPO_4_, pH 7.4), sonicated, and cleaned by centrifugation (3,500 *g* for 5 min, 4°C). The supernatant protein concentration was determined, as previously described (12, 13). Forty micrograms of protein were separated in a 4%–15% precast linear gradient polyacrylamide gel (Bio-Rad). After being transferred to nitrocellulose membranes, blots were rinsed with PBS and blocked in Odyssey blocking buffer in SuperBlock blocking buffer (Thermo Scientific) for 1 h at room temperature and incubated with rabbit polyclonal anti-connexin 43 (1:1,000, Cat. No. NB100–81867, NOVUS) overnight at 4°C. Total protein was used as a normalization control. Membranes were incubated with IR700-conjugated donkey anti-rabbit (1:1,000, Rockland Immunochemicals). Antibody labeling was visualized using Odyssey Infrared Scanner (LI-COR) for the detection of fluoroprobes, and fluorescence intensity analysis was performed using Odyssey software (LI-COR). Connexin 43 protein level was normalized using total protein in the samples.

### RNA Isolation and Real-Time PCR

Total RNA was isolated from LV tissues of male and female groups 7 days after MI surgery using TRIzol reagent (Invitrogen), and real-time qPCR was performed, as previously described (15). qPCR was performed in a 10 μL reaction mixture prepared with SYBR Green PCR Master Mix (Applied Biosystems, Warrington, UK) with diluted cDNA solution and 0.2 mM of each primer at 95°C for 10 min, followed by 35 cycles at 95°C for 10 s and 60°C for 45 s. All samples were run in triplicate, and the data were analyzed using the deltadelta Ct (ΔΔCt) method, with the *HPRT* gene serving as the normalization control. These transcripts were normalized to *Hprt1*. Primer sequences are described in Table 1.

### Histological Analysis and Infarction Size

Heart samples from male and female groups at *day 7* post-MI surgery were sectioned (5 lm) and stained using picrosirius red stain. LV infarct size was calculated by dividing the length of the infarcted area by the total circumference of the LV and expressed as a percentage.

### Statistical Analyses

Data are expressed as means ± standard error (SE). All data exhibited a normal distribution and comparable variability. The Shapiro–Wilk test was used to confirm the normal distribution of all data collected. Significant differences among groups were determined by two-way ANOVA with a Tukey post hoc test and unpaired Student’s *t* test followed by Bonferroni’s post hoc test when appropriate. The power analysis used in this study was based on multiple studies from our group, consideration of variance, power (1 — β = 0.80 probability levels with a two-tailed test at the α level = 0.05 probability of significance), and significance level (α). A *P* value of <0.05 indicates a significant difference. The estimated sample size per group was *n* = 5–10 animals with a large effect size (0.8–1.0).

## RESULTS

### Baseline Cardiac Function by Echocardiographic Assessment

**Figure 2.**
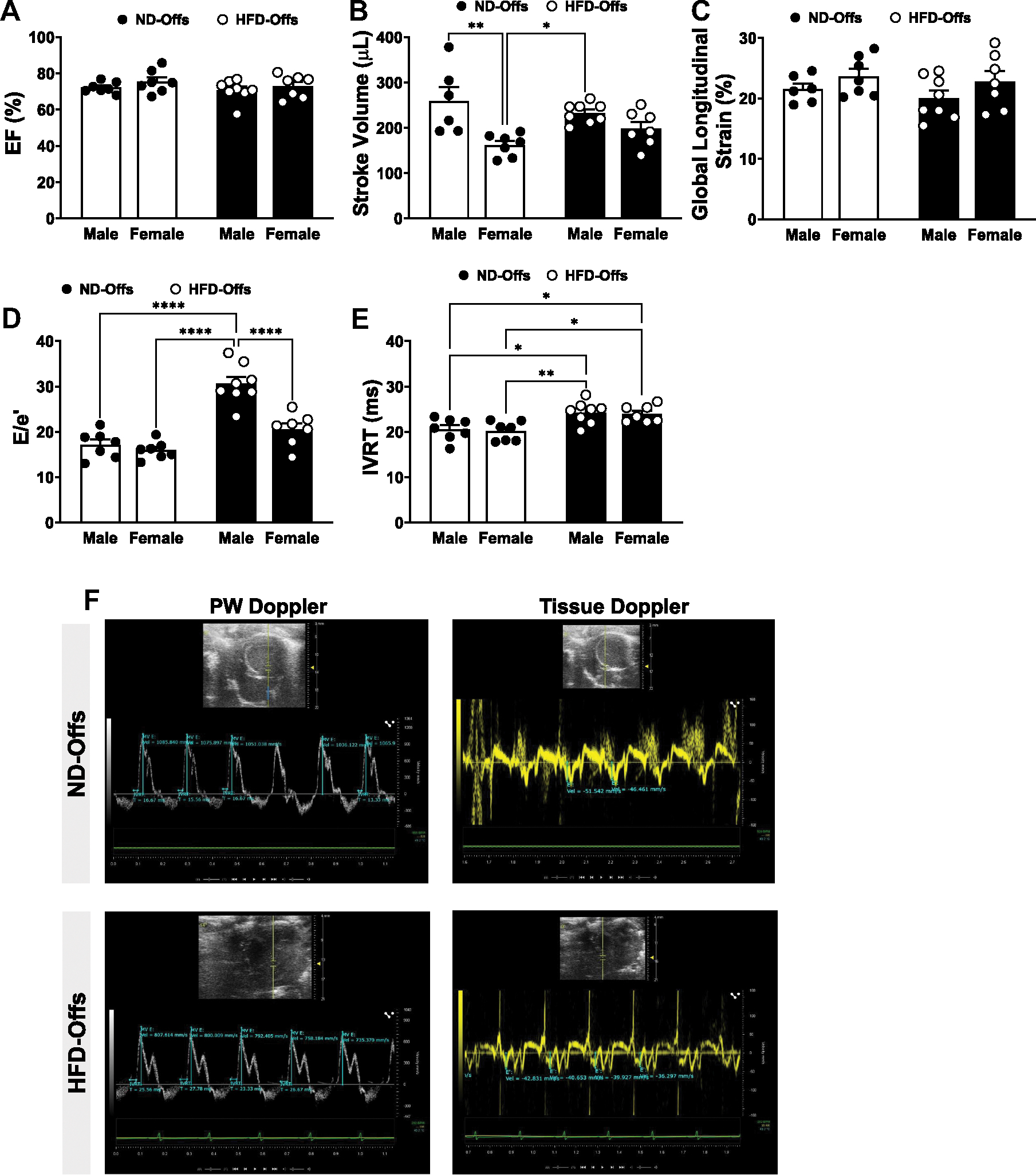
Cardiac phenotyping at 12 wk of age, before myocardial infarction, in male and female offspring from lean and obese parents. Ejection fraction (*A*), stroke volume (*B*), global longitudinal strain (*C*), E/e′ ratio (*D*), isovolumetric relaxation time (IVRT; *E*), and representative pulsed wave (PW) and tissue Doppler images (*F*). (*n* = 7 dams/group), *P < 0.05, **P < 0.01, ****P < 0.0001, two-way ANOVA with the Tukey post hoc test. HFD, high-fat diet; ND, normal diet.

Offspring born from obese parents exhibited similar systolic function as indicated by ejection fraction (EF) and global longitudinal strain when compared with offspring born from lean parents (Fig. 2, A and C) at 12 wk of age. Although no differences in stroke volume were observed between males and females, female ND-Offs showed lower stroke volume [sex, *P* = 0.004, *F*(1,24) = 16.69] compared with both groups of male offspring (Fig. 2B). Despite no differences in systolic function between groups, male and female HFD-Offs exhibited signs of diastolic dysfunction as indicated by a higher ratio of mitral valve (MV) early peak velocity (E) to tissue annular MV displacement e′ [E/e′; interaction, *P* = 0.0018, *F*(1,25) = 12.23; sex, *P* = 0.001, *F*(1,25) = 20.12; and groups, *P* < 0.0001, *F*(1,25) = 51.57] and isovolumetric relaxation time (IVRT) [groups, *P* = 0.0001, *F*(1,25) = 20.29] compared with male and female ND-Offs (Fig. 2, D–F).

### Impact of Myocardial Infarction on Survival Rate, Infarct Size, and Heart and Lung Weights in Offspring from Obese and Lean Parents

**Figure 3.**
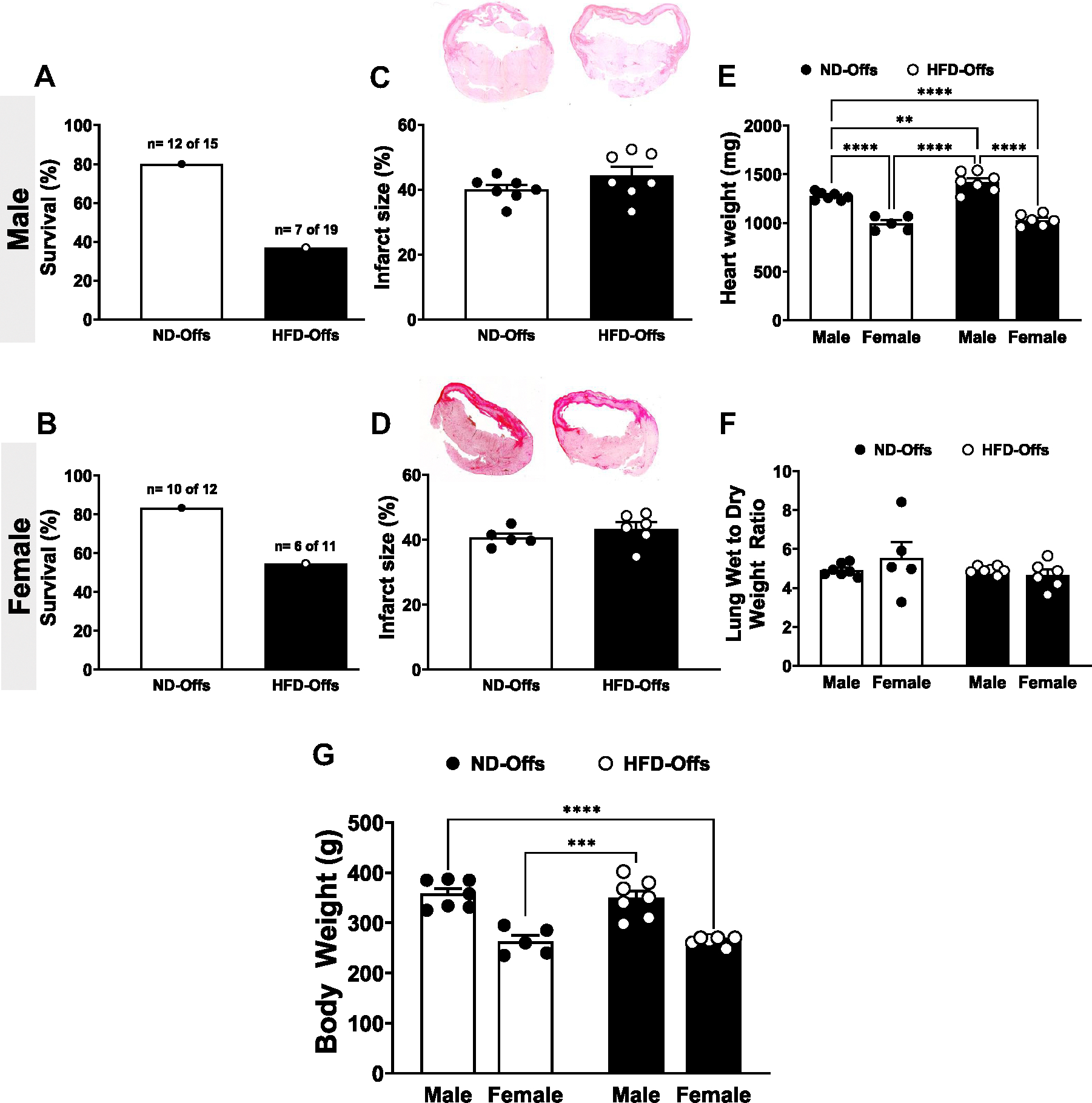
Survival rate, infarct size, and tissue weight 7 days postmyocardial infarction (MI) in male and female offspring from lean and obese parents, and offspring body weights before MI. Percent survival after MI (*A* and *B*), infarct size (*C* and *D*), heart weight (*E*), lung wet-to-dry weight ratio (*F*), and body weight before MI (*G*). (*n* = 7–10 dams/group, including survivors and nonsurvivors from MI surgery), **P < 0.01, ***P < 0.001, ****P < 0.0001, two-way ANOVA with the Tukey post hoc test. HFD, high-fat diet; ND, normal diet.

Male and female HFD-Offs born from obese parents exhibited reduced survival rates (males: 37% vs. 80%; females: 55% vs. 83% for HFD-Offs and ND-Offs, respectively, Fig. 3, A and B). The high mortality rate in male and female HFD-Offs was not associated with increased infarct size (Fig. 3, C and D), which was ~40% of the LV in both groups, and it was likely caused by ventricular arrhythmias, including VF occurring immediately after LAD ligation (observational data used to try to resuscitate the animals using cardiac massage but not quantified). In contrast, only one death was recorded after 24 h post-MI, which occurred 4 days post-MI surgery in male HFD-Offs. Heart weight was significantly higher in male, but not female, HFD-Offs when compared with same-sex ND-Offs [groups, *P* = 0.0030, *F*(1,22) = 11.16] (Fig. 3E). Expected heart weight differences between males and females were also observed in HFD-Offs and ND-Offs [sex, *P* < 0.0001, *F*(1,22) = 140.1] (Fig. 3E) and paralleled differences in body weight between males and females [sex, *P* < 0.0001, *F*(1,21) = 64.77] (Fig. 3G). No differences in wet-to-dry lung weight were observed among male or female groups (Fig. 3F). Thus, parental obesity is associated with an increased mortality rate in their offspring after induction of MI, even when offspring are fed a healthy diet after weaning and remaining lean.

### Cardiac Function in Response to Myocardial Infarction in Offspring from Obese and Lean Parents

**Figure 4.**
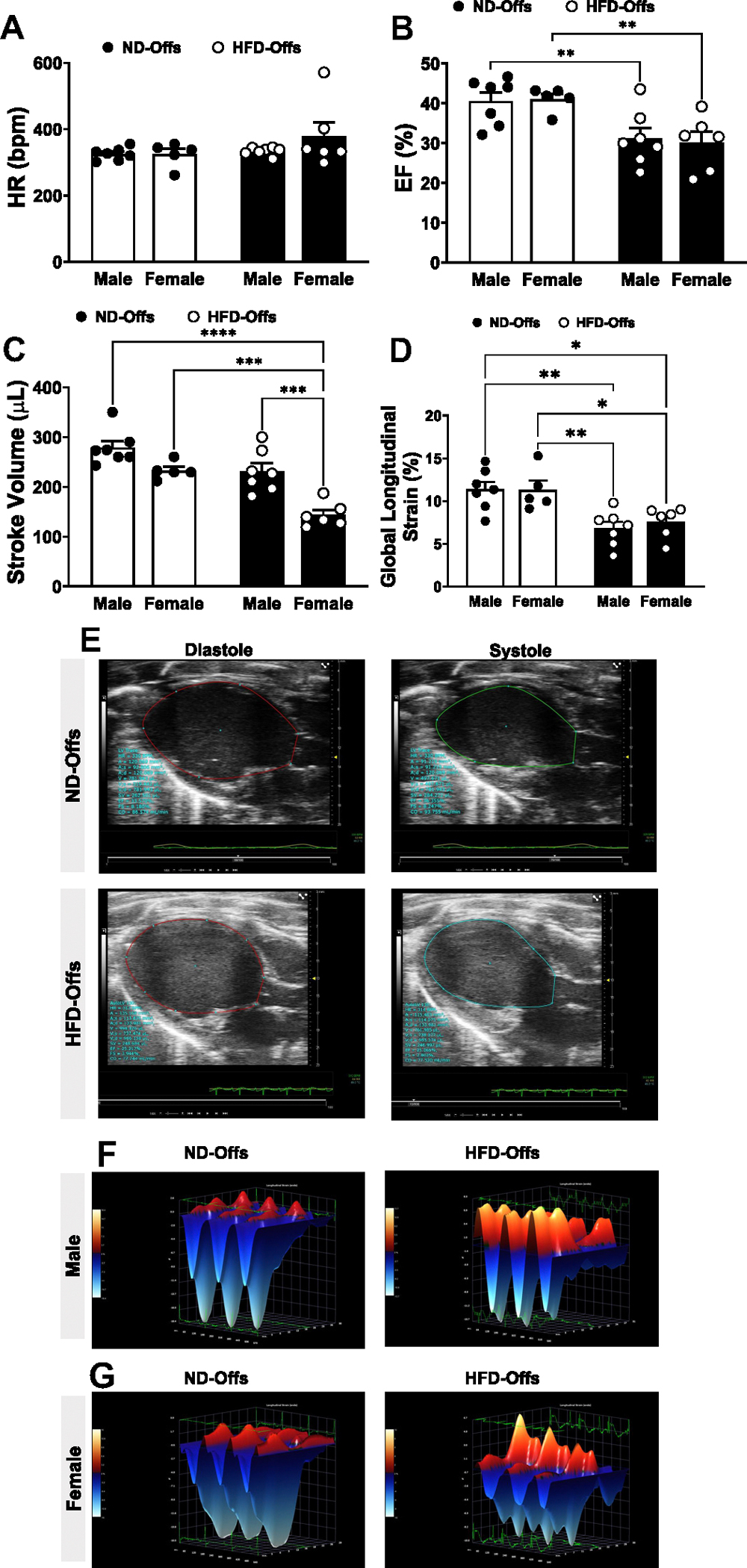
Echocardiographic measurements 7 days postmyocardial infarction in male and female offspring from lean and obese parents. Heart rate (HR; *A*), ejection fraction (EF; *B*), stroke volume (*C*), global longitudinal strain (*D*), representative B-mode long-axis left ventricle images (*E*), and three-dimensional representation of longitudinal strain in three consecutive cardiac cycles (*F* and *G*). (*n* = 5–7 dams/group), *P < 0.05, **P < 0.01, ***P < 0.005, ****P < 0.0001, two-way ANOVA with the Tukey post hoc test. HFD, high-fat diet; ND, normal diet.

**Figure 5.**
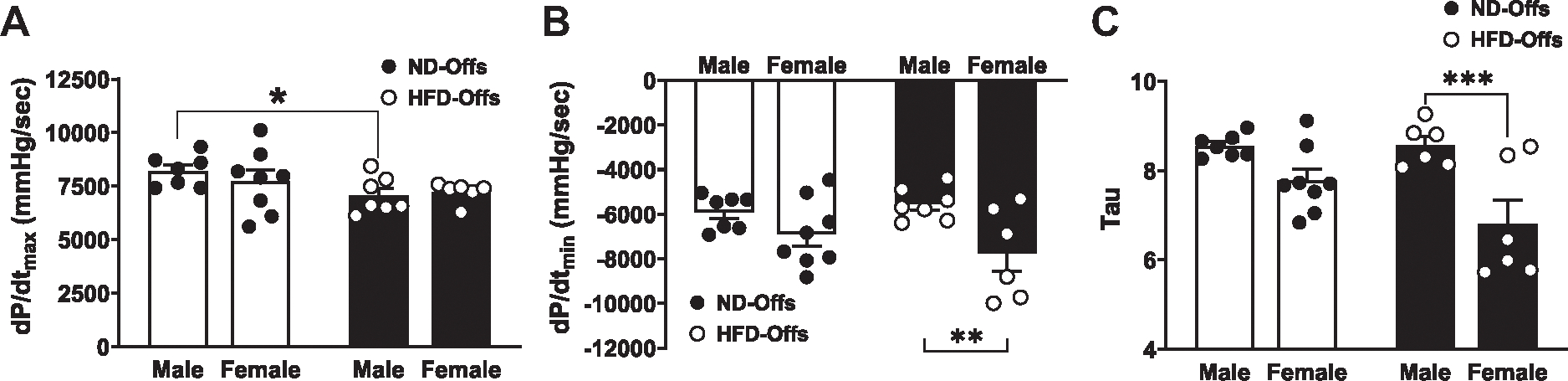
Intracardiac left ventricle function assessment 7 days postmyocardial infarction in male and female offspring from lean and obese parents. Maximal rate of left ventricle pressure rise (*A*), maximal rate of left ventricle pressure fall (*B*), and isovolumetric relaxation time constant (τ; *C*). (*n* = 5–7 dams/group), *P < 0.05, **P < 0.01, ***P < 0.005, two-way ANOVA with the Tukey post hoc test. HFD, high-fat diet; ND, normal diet.

Male and female offspring from obese parents exhibited similar heart rates compared with ND-Offs (Fig. 4A). However, male and female HFD-Offs showed more pronounced reductions in ejection fraction [groups, *P* = 0.0004, *F*(1,21) = 17.44] (EF, Fig. 4, B and E) and global longitudinal strain compared with ND-Offs [groups, *P* = 0.0001, *F*(1,22) = 21.73] (Fig. 4, B and D) despite similar infarct size among the groups (Fig. 3, C and D). Female HFD-Offs also exhibited reduced stroke volume compared with all other groups [sex, *P* < 0.001, *F*(1,21) = 25.97; groups, *P* < 0.001, *F*(1,21) = 27.03] (Fig. 4, C and E), whereas male HFD-Offs showed a tendency for reduced stroke volume compared with male ND-Offs (*P* = 0.0564). A three-dimensional (3-D) representation of longitudinal strain in three consecutive cardiac cycles in male and female rats is shown in Fig. 4, E and F. This 3-D representation indicates a more pronounced impairment of systolic function in HFD-Offs compared with ND-Offs. We also observed a significantly lower maximal rate of left ventricle pressure rise (d*P*/d*t*_max_) in male, but not female, HFD-Offs compared with ND-Offs [groups, *P* = 0.0438, *F*(1,24) = 4.4531] (Fig. 5A). The impact of MI on diastolic function (e.g., LV d*P*/d*t*_min_, and s), however, was similar between groups, and we only observed differences when comparing male versus female HFD-Offs [sex, *P* = 0.0046, *F*(1,24) = 9.755] (Fig. 5, B and C).

### Cardiac Metabolism in Response to Myocardial Infarction in Offspring from Obese and Lean Parents

**Figure 6.**
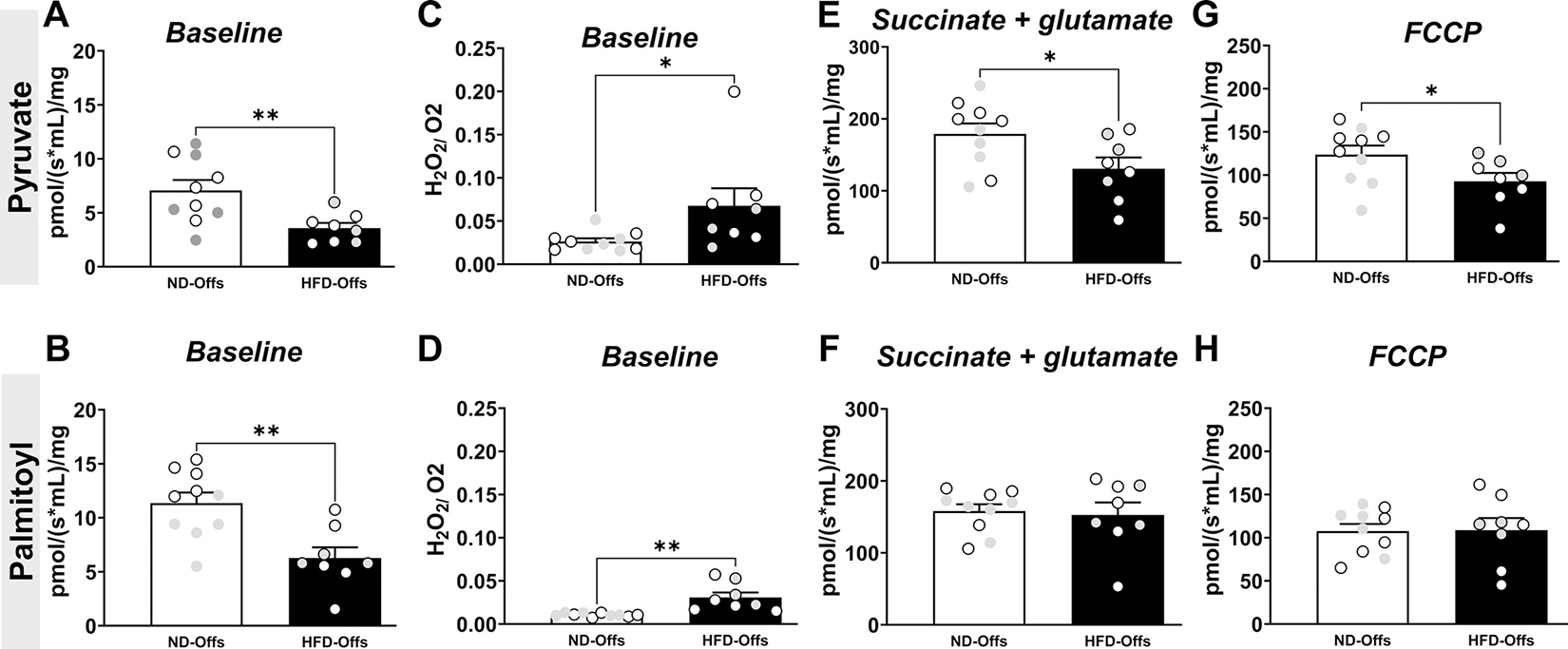
Mitochondrial function and metabolism in isolated cardiac fibers from male and female offspring from lean and obese parents 7 days postmyocardial infarction. Maximal oxygen consumption rate at baseline (*A* and *B*), H_2_O_2_ production/O_2_ consumption ratio (*C* and *D*), pyruvate-supported mitochondrial respiration rates to support direct electron flux complexes I and II of the electron transport chain (*E*), palmitoyl-supported mitochondrial respiration rates to support direct electron flux complexes I and II of the electron transport chain (*F*), and carbonyl cyanide 4-trifluoromethoxy phenylhydrazone (FCCP) maximal uncoupled oxygen consumption in isolated (*G* and *H*), permeabilized cardiac fibers of male (gray circles) and female (white circles) offspring from lean and obese parents. (*n* = 5–7 dams/group), *P < 0.05, **P < 0.01, unpaired two-tailed *t* test. HFD, high-fat diet; ND, normal diet.

Using high-resolution in situ respirometry, we assessed mitochondrial function in isolated cardiac fibers from the LV remote region (i.e., septum) and found reduced baseline oxygen consumption rate [pyruvate: *t*(16) = 3.026, *P* = 0.0080; palmitoyl: *t*(16) = 3.612, *P* = 0.0023] and increased mitochondria superoxide production [pyruvate: *t*(16) = 2.245, *P* = 0.0392; palmitoyl: *t*(16) = 3.948, *P* = 0.0012] in HFD-Offs at the state without adding ADP and substrates (Fig. 6, A–D). Males (gray circles) and females (white circles) were grouped together for mitochondrial function analysis since we did not observe significant differences between males and females. We found that cardiac fibers from HFD-Offs had reduced pyruvate-supported respiration rate when pyruvate was supplied in combination with malate, glutamate, and succinate, supporting electron flux via mitochondrial complexes I and II [pyruvate: *t*(16) = 2.269, *P* = 0.0375], whereas no differences between groups were observed for palmitoyl-supported respiration (Fig. 6, E and F). The maximal oxygen consumption rate during pyruvate-supported respiration, but not for palmitoyl carnitine-supported respiration, along with the maximal electron transport chain capacity (+ FCCP), was also significantly reduced in HFD-Offs compared with ND-Offs [pyruvate: *t*(16) = 2.137, *P* = 0.0484] (Fig. 6, G and H). These data suggest that parental obesity programs reduced carbohydrate metabolism in cardiac muscle mitochondria in their male and female offspring when offspring are submitted to MI.

### Impact of Parental Obesity on Connexin 43 Protein Expression and Genes Involved in Potassium and Calcium Regulation in Hearts from Offspring of Obese and Lean Parents

**Figure 7.**
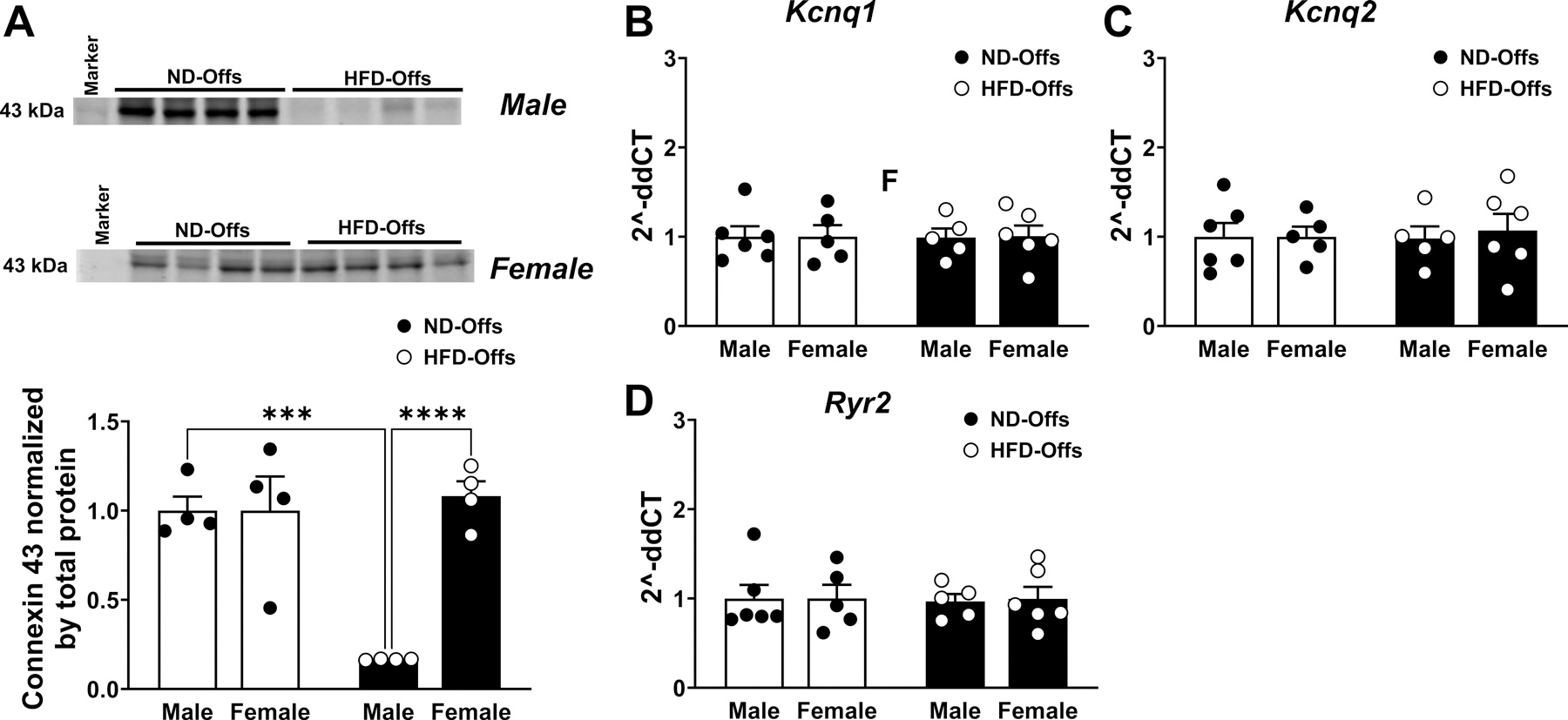
Cardiac connexin 43 protein levels in naive rats and potassium and calcium channels gene expression in heart samples 7 days postmyocardial infarction in male and female offspring from obese parents. Cardiac connexin 43 protein levels in male and female offspring not submitted to myocardial infarction (*A*), cardiac *Kcnq1* and *Kcnq2* mRNA levels (*B* and *C*), and cardiac *Ryr2* mRNA levels (*D*). (*n* = 4 or 5 dams/group), ***P < 0.005, ****P < 0.0001, two-way ANOVA with the Tukey post hoc test. HFD, high-fat diet; ND, normal diet.

We further investigated potential mechanisms responsible for increased mortality rate and worse systolic dysfunction in offspring from obese parents submitted to MI. We found that male, but not female, HFD-Offs had significant reductions in cardiac connexin 43 protein levels, the junction in the heart, in naive rats with no MI injury [interaction, *P* = 0.0014, *F*(1,12) = 16.98; sex, *P* = 0.0014, *F*(1,12) =16.98; groups, *P* = 0.0054, *F*(1,12) = 11.45] (Fig. 7A). We alsomeasured potassium voltage-gated channel subfamily Q members 1 and 2 (*Kcnq1* and *2*) and the ryanodine receptor type 2 (*Ryr2*) gene expressions in LV samples collected 7 days post-MI and found no significant differences between male or female HFD-Offs when compared with ND-Offs (Fig. 7, B–D).

## DISCUSSION

This study tested the hypothesis that parental obesity programs their offspring to worse outcomes after MI and examined potential mechanisms involved, including altered mitochondrial metabolism and expression of genes involved in the propagation of electrical impulses in the heart. Our results indicate that, despite being maintained on a normal diet throughout their lives, male and female offspring from obese parents showed signs of diastolic dysfunction in adulthood as well as a reduced survival rate and worse systolic dysfunction after MI when compared with offspring from lean parents. In addition, parental obesity was associated with reduced LV expression of connexin 43, a key protein for cardiac myocyte propagation of electrical impulses, in male but not female offspring. These findings suggest that parental obesity may worsen cardiac outcomes and increase mortality following MI in male and female offspring, although there may be significant sex differences in the potential mechanisms involved.

### Obesity and Cardiovascular Disease Risk

Obesity and overweight are major health challenges in the United States, with childhood rates of obesity steadily increasing. Current evidence suggests that children of parents with obesity often become obese, although it has been challenging to distinguish environmental factors involved from developmental and genetic/epigenetic contributors (16, 17). Data from multiple studies in humans show that obesity is also associated with an increased risk for cardiovascular diseases, including heart failure. For instance, the Framingham Heart Study demonstrated that for each kg/m^2^ increase in body mass index (BMI), the risk of heart failure increased by 5% in men and 7% in women (18). In addition, heart failure develops 10 years earlier in individuals with obesity compared with those with normal BMI (18). Twenty years of obesity increases the prevalence of heart failure by 70%, and after 30 years of obesity, the prevalence rises by 90% (19). The longer obesity and metabolic disorders persist, the greater the risk of developing cardiovascular and metabolic diseases. Thus, preventing obesity and providing early treatment are essential for reducing the risk of heart failure later in life.

### Impact of Parental Obesity on Offspring Cardiac Function

Although it is clear that obesity increases the risk of cardiac dysfunction and heart failure, and considering the steady increase in rates of obesity/overweight in young adults at childbearing ages, parental obesity may also become an important risk factor for cardiovascular diseases in their offspring. Therefore, we hypothesized that parental obesity increases offspring susceptibility to cardiac dysfunction in response to stressors during adult life, such as acute MI, even when the offspring are maintained on a normal healthy diet and do not become obese. Our findings confirmed our hypothesis and indicated that parental obesity programs their offspring to exacerbate cardiac dysfunction and increase mortality after MI even when the offspring are fed a normal diet and never become obese.

We previously demonstrated that parental obesity contributes to diastolic, but not systolic, dysfunction in 1- to 3-day-old offspring in a sex-dependent manner (12). Although male and female offspring from obese parents exhibited similar metabolic alterations, including increases in body weight, blood glucose, and insulin levels, male offspring from obese parents also exhibited early signs of diastolic dysfunction that were associated with mitochondrial dysfunction (12). These findings underscore significant sex differences in the impact of parental obesity on cardiac function early in life. In the present study, however, we found that at adulthood, nonobese males as well as female offspring from obese parents exhibited signs of diastolic dysfunction, whereas their baseline systolic function, before MI, remained similar to that of male and female control off-spring from lean parents.

### Impact of Parental Obesity on Offspring Cardiac Function in Response to MI

Previous studies showed that the offspring of overweight mothers are at increased risk of long-term cardiovascular complications, including hypertension, coronary artery disease, stroke, and heart failure (3). Calvert et al. (20) found that maternal obesity is linked to increased myocardial ischemia-reperfusion injury in obese agouti (A^y^) mice (20). Our results demonstrate that parental obesity is associated with worse cardiac outcomes after MI even when the off-spring remain lean. Moreover, we found that male and female HFD-Offs exhibit worse systolic dysfunction and higher mortality rates compared with ND-Offs despite no significant difference in infarct size between the groups. These observations suggest that worse cardiac outcomes observed in offspring from obese parents cannot be explained by a larger area of ischemia resulting from the MI procedure.

Despite worse cardiac dysfunction in offspring from obese parents, there were no significant differences in lung wet-to-dry weight ratio between groups. One potential explanation for these findings is that we assessed cardiac function and lung congestion at an early stage of post-MI remodeling (7 days after MI surgery). This early phase is characterized by myocardial deformation and remodeling of the myocardium (21) and may be too early to detect pulmonary congestion in our model. Future studies are needed to explore the long-term consequences of parental obesity on MI-induced congestive heart failure and pulmonary edema.

### Mechanisms Underlying Cardiac Dysfunction in Offspring from Obese Parents

Although the precise mechanisms responsible for increased cardiac dysfunction and greater mortality after MI in off-spring from obese parents are still unclear, we found that male, but not female, HFD-Offs exhibit lower LV connexin 43 protein levels compared with ND-Offs controls at baseline before MI. Yang et al. (22) suggested that connexin 43 under-goes structural remodeling and redistribution following MI, and these alterations affect intercellular communication and electrical conduction, leading to increased susceptibility to postinfarction VA. Previous studies showed abnormal connexin 43 expression in several forms of cardiomyopathies, including ischemic cardiomyopathy (23). Thus, further experiments are needed to test whether potential changes in connexin 43 expression following MI are influenced by parental obesity. Nevertheless, our observation that ventricular arrhythmias mostly occurred within minutes after LAD ligation to induce MI suggests that basal connexin 43 levels before MI would likely be more important in contributing to alterations in cardiac currents than changes in protein levels elicited by MI.

It is also possible that underlying diastolic dysfunction in offspring from obese parents, when combined with an additional cardiac stressor (i.e., acute MI) in adulthood, may have interacted additively or synergistically to induce a more severe phenotype characterized by poor survival and profound cardiac impairment. We also observed more VF, beginning within 5–10 min after LAD occlusion, in male and female HFD-Offs compared with ND-Offs, which likely also contributed to higher mortality in offspring from obese parents. Thus, our findings support the hypothesis that pre-existing diastolic dysfunction, when compounded by superimposed cardiac stress, may accelerate the progression of cardiac dysfunction and partially explain the increased cardiac vulnerability to stress in offspring from obese parents.

Another potential mechanism contributing to worse cardiac dysfunction after MI in lean offspring from obese parents could be exaggerated inflammatory responses. This may involve local production and release of cytokines and chemokines, recruitment of innate immune cells, and impaired resolution of inflammation post-MI surgery. This possibility is supported by our previous findings showing that male off-spring from obese parents exhibit greater inflammatory cell infiltration in the kidney cortex and medulla in response to renal ischemia/reperfusion injury (24). However, a limitation of the present study is that we did not assess cardiac inflammation following MI. Future studies are needed to examine the potential role of exacerbated cardiac inflammatory response in contributing to worse cardiac outcomes after MI in offspring from obese parents.

### Parental Obesity and Offspring Cardiac Mitochondrial Function Post-MI

In heart failure, the heart experiences an energy deficit primarily due to a reduction in mitochondrial oxidative capacity, which is partly compensated by increased reliance on ATP production via glycolysis. The relative contribution of various fuels for mitochondrial ATP production shifts as the disease progresses. For instance, the oxidation of glucose and amino acids decreases, ketone oxidation increases, and fatty acid oxidation may either increase or decrease depending on the specific type of heart failure (25). We found reduced myocardial mitochondrial capacity to metabolize carbohydrates in male and female offspring from obese parents submitted to MI, whereas mitochondrial capacity to metabolize long-chain fatty acids was similar to that observed in cardiac fibers of control offspring from lean parents. Previous studies also suggest that under hypoxia and ischemia, the aerobic oxidation of fatty acids decreases, whereas glycolysis increases, shifting glucose to become the main energy substrate for myocardial metabolism (26). In addition, other studies demonstrated that enhancing glucose oxidation during reperfusion is closely associated with reduced ischemia/reperfusion injury and improved cardiac function (27–29). Thus, impaired myocardial oxidative carbohydrate metabolism may also contribute to worse cardiac outcomes after an acute MI in offspring from obese parents.

In the present study, we also found a reduced oxygen consumption rate and increased superoxide production in the cardiac fibers of offspring from obese parents. Previous studies showed that reactive oxygen species, including superoxide, contribute to the progression of cardiac dysfunction after MI (30), and scavenging superoxide reduces proinflammatory cytokines and collagen isoforms, resulting in improvement of cardiac function 21 days after a 30-min ischemia followed by reperfusion (31). Therefore, impaired mitochondrial function (i.e., reduced carbohydrate metabolism and increased superoxide) is another potential factor contributing to the poor outcomes observed in off-spring from obese parents. However, whether increased superoxide production is a cause or consequence of the worse cardiac dysfunction observed in our model is unclear and beyond the scope of the current study.

We previously showed that SIRT3, a major regulator of mitochondrial enzyme deacetylation that plays an important role in regulating mitochondria energy production and cardiac remodeling in heart failure, is reduced in the hearts of male HFD-Offs as early as 21–23 days after birth (12). We also found reduced cardiac mtFA expression, an index of mitochondria DNA content, in male HFD-Offs (12). These findings also help explain, at least in part, why male off-spring from obese parents exhibit a worse survival rate after MI in offspring from obese parents.

### Summary and Perspective

Our findings indicate that parental obesity is linked to diastolic dysfunction, increased mortality rate, and worse systolic dysfunction after induction of MI in their offspring, even when offspring are fed a healthy diet postweaning and never become obese. Notably, the impact of parental obesity on offspring susceptibility to worse outcomes when facing a cardiac stressor such as MI was not affected by the sex of the offspring, although potential mechanisms appear to be heavily sex-dependent. Overall, these findings highlight the importance of preventing/reducing obesity in couples who plan on conceiving to mitigate potential adverse cardiovascular consequences in future generations.

## Figures and Tables

**Table 1.** qRT-PCR primers

Gene	Species	Forward	Reverse

*Kncq2*	Rat	5'-GATCGCCTTCTACCGGAAAG-3'	5'-TCCTTCTCCATCACCACTTCA-3'
*Ryr2*	Rat	5'-GACCATACATCCTGCCTCTAAG-3'	5'-CCGTATGACAAGTGCAAGTACC-3'
*Kcnq1*	Rat	5'-ACCGTCTTCCTCATTGTTCTG-3'	5'-GACCACATACTCTGTCCCAAAG-3'
*Hprt*	Rat	5'-CCCCAAAATGGTTAAGGTTGC-3'	5'-AACAAAGTCTGGCCTGTATCC-3'

## Data Availability

Data will be made available upon reasonable request.
